# Management of second metacarpal chronic osteomyelitis by induced membrane technique

**DOI:** 10.1080/23320885.2018.1489250

**Published:** 2018-08-07

**Authors:** William Tabib, Hanna Haddad

**Affiliations:** Department of Orthopaedic and Trauma Surgery, Centre hospitalier de Tulle, Tulle, France

**Keywords:** Induced membrane, osteomyelitis, metacarpal, bone repair

## Abstract

Management of chronic osteoarticular infection of the hand is a true challenge. Several methods of treatment are described in the literature to eradicate the infection. We present a case in which chronic osteomyelitis of the second metacarpal was treated by induced membrane technique, with successful outcome and complete hand recovery.

## Introduction

The induced membrane technique was originally used in the treatment of bone loss from the long bones. However, in recent years it has increasingly been used to treat the loss of osteoarticular matter from the hand and wrist.

Regardless of the location, the technique is the same and involves a two-stage surgical procedure.

In the first stage, segmental bone resection with infectious tissue debridement and cement spacer interposition are performed, combined with stable fixation.

In the second stage, a cancellous bone graft is placed in the biological chamber induced by the cement spacer.

Despite the apparent simplicity of the technique, the technical procedures recommended by Masquelet [1] must be carefully followed.

The literature review found several cases in which this technique was used to treat complex trauma of the hand, but very few involved osteoarticular infection of the hand. This will be illustrated by a case study.

## Case

Mr. BH, 39 years old, right-handed, presented with a Bennett fracture of the first right metacarpal following a work accident. The fracture was initially treated in another institution by reduction and percutaneous pinning using Iselin’s technique. Three weeks later, the patient developed an inflammatory reaction around the entry point of the distal pin, at the second metacarpal. A purulent discharge led to early removal of the distal pin. The isolated organism was a methicillin-sensitive Staphylococcus aureus. Regular topical treatment was given, and antibiotic therapy was initiated for five weeks based on laboratory results. Bone healing of the Bennett fracture was obtained in the sixth week after the accident, and the proximal pin was removed.

**Figure 1.**
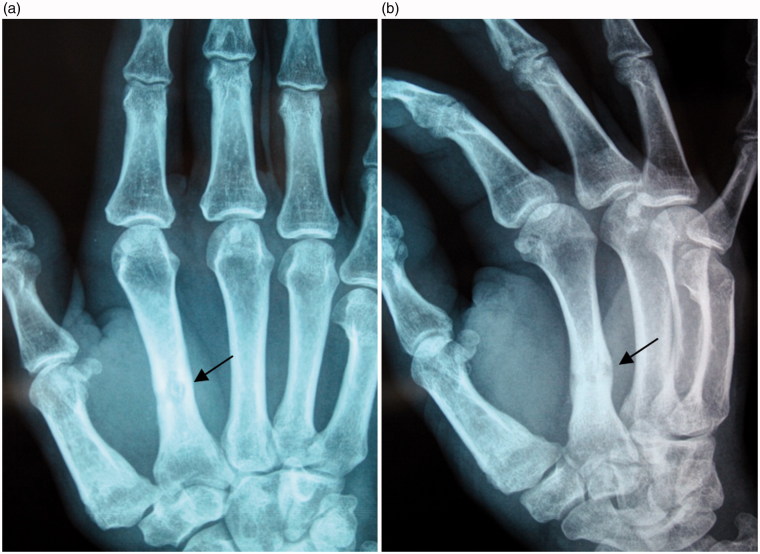
(a,b) Standard X ray of the hand: the black arrow shows the focus of osteomyelitis corresponding to the path of the distal Kirschner wire previously used to treat the Bennett fracture.

**Figure 2.**
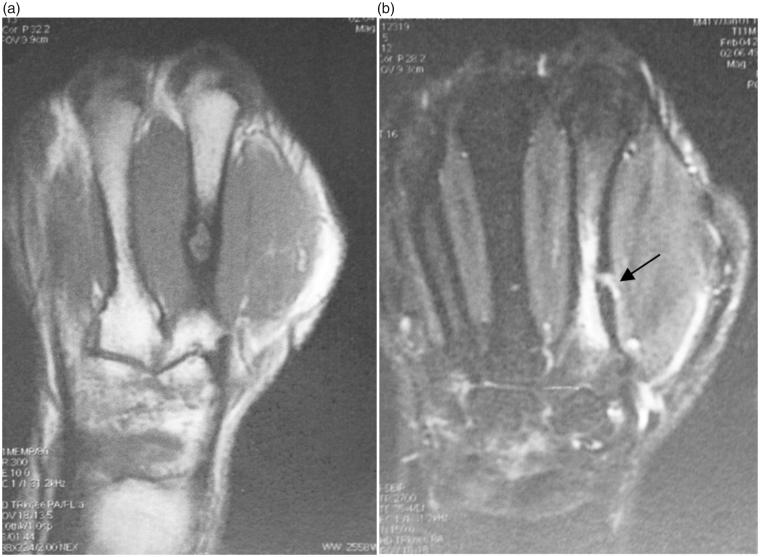
(a,b) MRI of the hand shows an intraosseous second metacarpal geode and the path of the fistula indicated by the black arrow.

Three months later, the patient was referred to us by his doctor because of persistent pain at the second right metacarpal, accompanied by attacks of inflammation and intermittent purulent discharge. Standard hand X-ray demonstrated the presence of an area of osteolysis surrounded by osteocondensation at the second metacarpal, indicating a focus of osteomyelitis along the path of the distal pin (Figure 1). An MRI was performed on the hand. It confirmed the diagnosis, revealing the extent of the focus of osteomyelitis, the path of the fistula, and the inflammatory extension into neighboring soft tissues (Figure 2).

Surgical treatment called for the induced membrane technique.

The first stage included a segmental resection of the area of osteomyelitis through a dorsal approach with excision of the reformed tissue while preserving the index finger extensor tendon.

**Figure 3.**
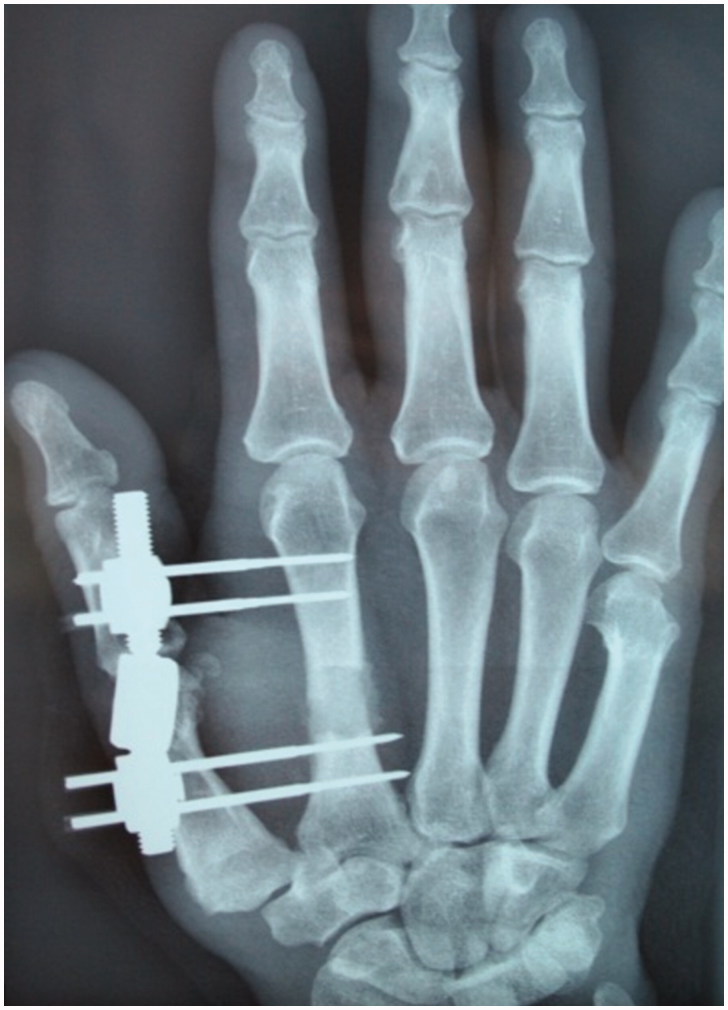
First stage post-operative X- ray control after segmental resection, cement spacer interposition and stabilization with mini external fixator.

The second metacarpal was stabilized with a mini external fixator, and a cement spacer was interposed (Figure 3).

Antibiotic therapy was initiated for six weeks based on sensitivity testing. The external fixator was then removed, and the hand temporarily immobilized with a splint until there was healing along the path of the inserts.

**Figure 4.**
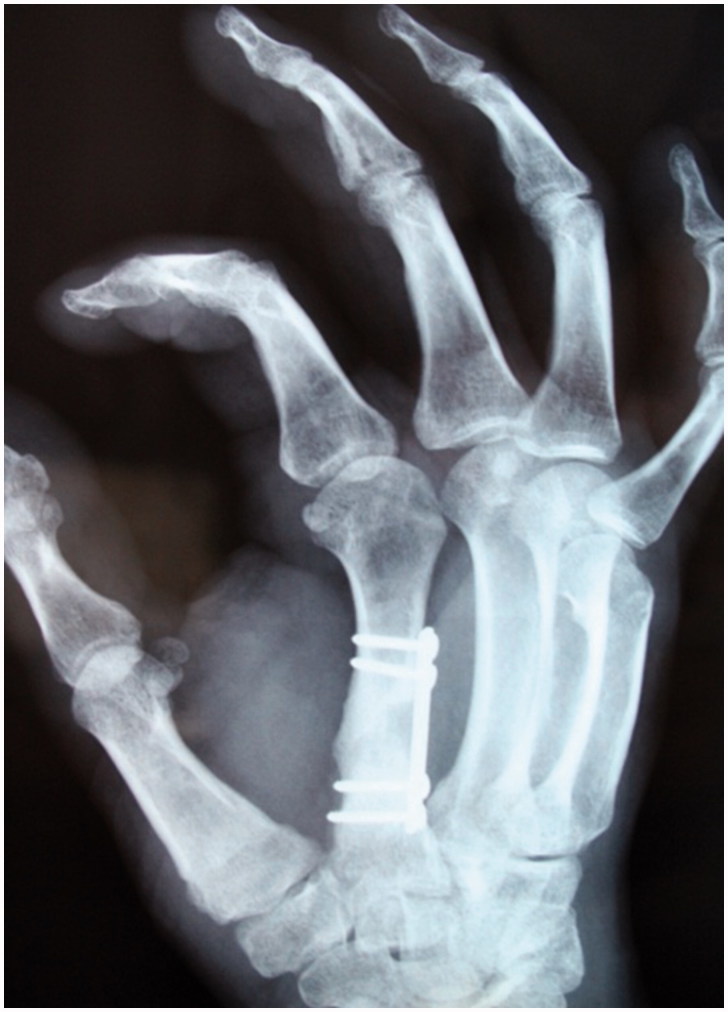
X ray control three months after the second stage, showing consolidation of the bone graft.

**Figure 5.**
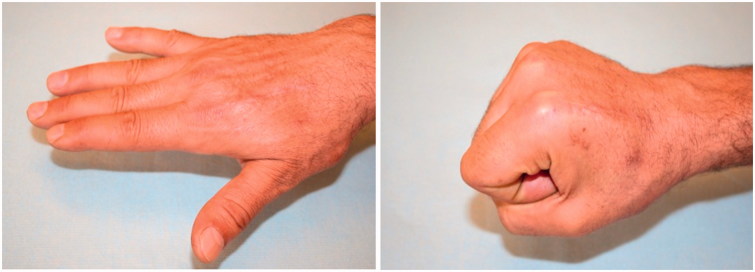
Shows full recovery of hand function.

**Figure 6.**
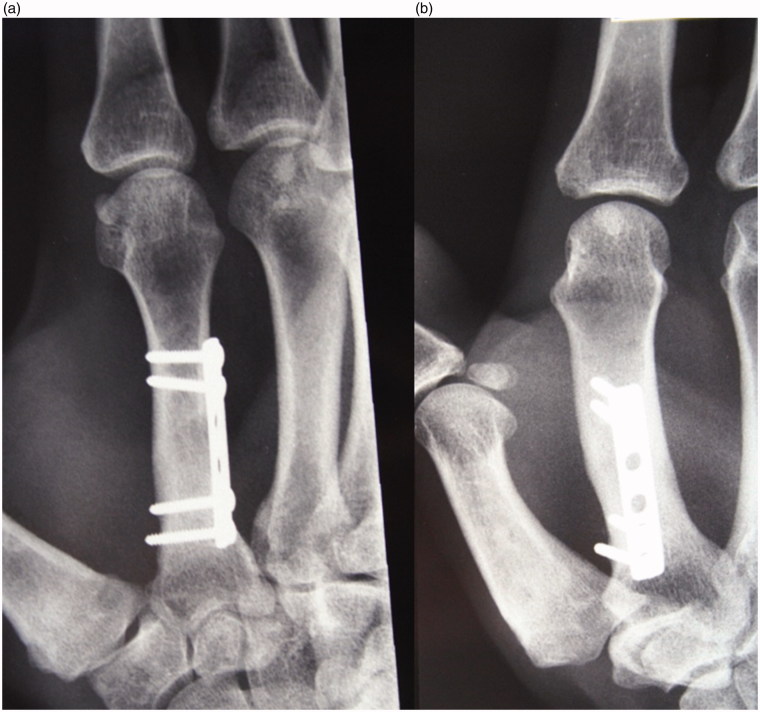
(a,b) X- ray at 36-month follow-up shows complete incorporation of the graft with bone corticalization.

The second stage was performed in the eighth week. The cement spacer was removed and the biological chamber was filled with a spongy bone graft taken from the ipsilateral iliac crest. Internal fixation was performed with a mini plate while preserving the induced membrane. The postoperative period was uneventful. Bone healing was observed three months later (Figure 4) with complete disappearance of pain. The patient has regained normal use of the hand with a Quick DASH score of 11 and complete mobility (Figure 5). We are currently at the 36-month follow-up. X-ray of the hand shows incorporation of the graft and evidence of corticalization (Figure 6).

## Discussion

The induced membrane technique was initially described by Masquelet [1] for the reconstruction of losses from the diaphyses of the long bones, whether from trauma, infection or after tumor surgery. Pelissier et al. [2] demonstrated the role of the induced membrane in the secretion of growth factors involved in bone healing: vascular endothelial growth factor (VEGF), transforming growth factor beta-1 (TGFB1) and osteo-inducing factors such as bone morphogenic protein-2 (BMP2).

Masquelet et al. [3] highlighted the possibility of extending the application of this technique to treating bone losses in the hand.

A review of the literature found the first clinical applications in the hand were undertaken in the 2000s, with two metacarpal reconstructions reported by Ribière et al. [4].

In 2004, Proubasta et al. [5] reported on a case in which osteoarticular infection of the third finger was successfully treated with this technique.

Two other short published series supported this data.

The first, reported by Flamans et al. [6], was a prospective study of 11 cases of loss of skeletal bone matter from the hand. The series was divided into eight cases of open fracture of the fingers with loss of bone and integumentary matter, and three cases of secondary management of osteoarticular infection (thumb, wrist and fifth finger). The mean follow-up time of the study was 20 months, with nine successes and two failures. The failures were attributed to exposure to the cement spacer in one case, and a technical error in the other case. The average healing time after the bone graft in the nine successful cases was 4.1 months.

The second series, reported by Mure et al. [7], involved seven cases of loss of bone matter following complex trauma to the hand. This was a prospective study with a mean follow-up time of 23 months. Bone healing was achieved in all cases. There were no cases of chronic infection in this series.

Ardouin et al. [8] reported on a case of rescue of the vascularized transfer of a hallux to a thumb, which was complicated by infection. Again, application of the two-stage Masquelet technique produced a good final result.

More recently, Moris et al. [9] reported successful application of the technique to several metacarpal segments of a traumatized hand. In a retrospective assessment, Moris et al. [10] also reported the functional and X-ray results of a series of 18 patients treated for the loss of bone matter from the hand and wrist, with 16 successes and two failures.

Analysis of this series did not find any cases of osteoarticular infection. Our observation is distinguished by the fact that it involved a bone infection secondary to a pinning for a closed Bennett fracture. Inflammatory reactions around osteosynthesis pins are not uncommon in the hand and wrist. They are generally superficial and have a favorable outcome after removal of the pin and topical treatment. The occurrence of chronic osteomyelitis remain difficult complication to treat.

In our case, the second stage was performed eight weeks after the first. The work of Pelissier et al. [2] in animals demonstrated that the ideal time to perform this second stage was the fourth week when the BMP2 secretion rate was at a maximum. The same finding is reported in the works of Aho et al. [11].

In clinical practice, analysis of the different observations reported in the literature revealed that this second stage was carried out between the sixth and eighth week. This is accepted as the normal timescale by various authors [3,6,7], due to time necessary for antibiotic therapy and local healing.

In our case, after removal of the cement spacer, the area of bone matter loss was filled with an iliac graft. However, bone grafts taken from the radius, or composite grafts have previously been used successfully on the hand [7,12].

The simplicity and reproducibility of the technique, the possibility of applying it at any age, and the lack of any specific ancillary equipment led us to prefer this reconstruction procedure over other traditional graft or vascularized graft techniques, the latter being technically more demanding. The protective role of the pseudo-synovial membrane vis-à-vis the bone graft has been well recognized by various authors [3,6].

The induced membrane technique is an alternative to other bone reconstruction techniques. Although simple and accessible, this procedure must comply with the technical procedures detailed by Masquelet. Its application in osteoarticular infection of the hand is still not very widespread. It opens up new therapeutic possibilities in hand surgery, which should be assessed on a larger number of patients.
